# Interferons and STAT1 dynamically modulate integrated stress response outcomes in CD8 T cells

**DOI:** 10.21203/rs.3.rs-10692330/v1

**Published:** 2026-09-04

**Authors:** Rachael Philips, Kang Yu, Yi-Chu Liao, SeolKyoung Jung, Kan Jiang, Yuka Kanno, Davide Randazzo, Scott Oakes, John O’Shea

**Affiliations:** National Institutes of Health; NIAMS/NIH; NIAMS/NIH; National Institute of Arthritis, Musculoskeletal, Skin, NIH; National Institutes of Health; NIH / NIAMS; NIAMS/NIH; University of Chicago; National Institutes of Health

## Abstract

Type I interferons (IFN-I) drive both protective and pathological CD8 T cell responses. Patients with activating STAT1 mutations exhibit enhanced IFN responses with T cell dysfunction and autoimmunity; however, mechanisms underlying excess versus impaired response remain unclear. We used mouse models expressing a STAT1 mutation (*Stat1*-T385M) ubiquitously or T-cell selectively to dissect T cell-intrinsic versus cell-extrinsic actions of activating STAT1. Global *Stat1*-T385M expression had increased lethality upon acute viral infection with CD8 T cell dysfunction marked by stress response activation rather than classical exhaustion. This dysfunction was extrinsic, as T-cell-restricted *Stat1*-T385M preserved expansion, cytokine production, and checkpoint receptor expression. However, intrinsic *Stat1*-T385M reshaped differentiation by suppressing T-bet and effector programs while upregulating a stress signature seen in memory lymphocytes. The extrinsic-intrinsic switch depended on IFN-I magnitude, with exaggerated levels triggering a pro-apoptotic stress response. Therefore, STAT1 works as a rheostat balancing CD8 differentiation and stress, as a basis for interferonopathies.

Type I interferons (IFN-Is) have a dual role in antiviral CD8 T cell immunity, with the timing and level of IFN exposure determining CD8 T cell outcomes^1,2^. Early and acute IFN-Is directly enhance the expansion, effector differentiation, and functionality of CD8 T cells^3,4^, whereas persistent IFN-I signaling during chronic viral infection enables the transition of CD8 T cells into an exhausted state^5–8^. Signal transducers and activators of transcription 1 (STAT1) is a critical factor downstream of IFN-Is and has intrinsic anti-proliferative and pro-apoptotic consequences in naïve and early activated CD8 T cells^9–12^. Hence, STAT1 expression is downregulated during an effector response and IFN-Is signal through other STATs to enable their pro-survival and pro-proliferative actions^9–12^. What remains unclear is how protracted IFN-I and aberrant STAT1 activity during CD8 T cells effector response disrupts this dynamic during acute viral infection.

Patients with STAT1 gain-of-function (GOF) mutations have aberrant interferon signaling yet paradoxically suffer from multiple forms of autoimmunity along with recurrent and severe viral infections^13^. Mouse models harboring STAT1 GOF variants revealed an immunodeficiency after infection. We recently showed that innate lymphocytes from *Stat1* GOF mice fail to produce IFN-γ early after mouse cytomegalovirus (MCMV) infection, which later elicits hypercytokinemia and lethality^14^. Prior literature also indicates a CD8 T cell defect^15^; however, the mechanisms of *Stat1* GOF underlying CD8 T cell dysfunction remain obscure.

Using a conditional knock-in *Stat1* GOF model, we explored cell intrinsic and extrinsic roles of STAT1 in CD8 T cells beyond its recognized anti-proliferative and pro-apoptotic function. Here, we show that STAT1 functions as a dynamic regulator for CD8 T cell differentiation and stress-associated dysfunction. After acute viral infection, intrinsic *Stat1* GOF did not impede proliferation but instead repressed terminal effector differentiation and induced a memory-associated stress gene signature. However, global *Stat1* GOF expression led to excessive IFN-I programs that triggered a *bona fide* integrated stress response and cell death in CD8 T cells. These findings indicate that STAT1 GOF mutations confer heightened vulnerability to detrimental IFN-stress-induced outcomes, with implications extending to a broader spectrum of interferonopathy-associated disorders.

## RESULTS

### CD8 T cells from *Stat1*^T385M/+^ mice are dysfunctional after acute viral infection

Despite IFNs positive effect on lymphocyte activation, chronic IFN suppresses CD8^+^ T cell activity^5,6^. STAT1 is essential for IFN signaling, therefore we sought to examine the impact of STAT1 in mice expressing the activating mutation *Stat1-*T385M in a *Cre*-dependent manner^14^. We infected mice with LCMV-Armstrong (referred to LCMV-Arm), as a paradigmatic acute viral infection model dependent on CD8 T cells^16,17^. Mice that model patients were used first, where the *Stat1*-T385M allele was expressed ubiquitously (via *CMV*-cre) in a heterozygous manner (designated *Stat1*^T385M/+^, light orange throughout). Ordinarily, WT mice display no adverse effects, however, LCMV-Arm infection of *Stat1-*T385M mice resulted in viral lethality (Fig. 1a)^14^. Examination of total and GP33-specific *Stat1*^T385M/+^ CD8 T cells during infection revealed drastically impaired expansion (Fig. 1b–e). CD8 T cell differentiation was altered, in that antigen-specific CD8 T cells from *Stat1*^T385M/+^ mice exhibited fewer KLRG1^+^IL-7R^−^ short-lived effector cells compared to WT T cells day 7 post infection (p.i.). Additionally, memory precursor cells increased day 15 p.i. (Fig. 1f,g) compared to WT cells. Moreover, cytolytic terminal effector cells identified by CX3CR1 and KLRG1^18^ were reduced in *Stat1*^T385M/+^ mice compared to WT mice (Fig. 1h). Functionally, GP33-specific CD8 T cells from *Stat1*^T385M/+^ mice also presented with elevated expression of the immune checkpoint receptors PD-1, LAG3, TIGIT, and Tim3 (Fig. 1i,j) and impaired IFN-g production (Fig. 1k) compared to WT GP33-specific CD8 T cells. In sum, CD8 T cells from *Stat1*^T385M/+^ mice are dysfunctional during acute viral infection.

### CD8 T cells from *Stat1*^T385M/+^ mice are not canonical terminally exhausted

To identify detailed transcriptomic features of CD8 T cells after LCMV-Arm infection, we performed single cell RNA sequencing coupled with TCR sequencing (scRNA+TCR-seq) focusing on 7 days p.i. (Fig. 2a and Extended Data 1a). Consistent with our flow cytometry data (Fig. 1h), *Stat1*^T385M/+^ GP33-specific CD8 T cells exhibited dramatic reduction in KLRG1^+^CX3CR1^+^ Terminal Effector cells (blue, Fig. 2b and Extended Data 1b-c) and a relative increase of interferon-stimulated gene (ISG) and Cycling cells (purple and green, Fig. 2b). One replicate showed fewer Intermediate cells (light blue) and, notably, clustering analysis did not generate a distinct exhaustion population in *Stat1*^T385M/+^ mice (Fig. 2a,b; Extended Data 1a,b). Also, compared to the Tim3^+^PD-1^+^ exhausted population seen with LCMV clone 13^19,20^, *Stat1*^T385M/+^ GP33-specitic T cells lacked those features (Fig. 2c,d). These findings were further supported by GSEA analysis of antigen specific CD8 T cells in which low enrichment of terminal exhaustion gene signature were noted for *Stat1*^T385M/+^ GP33-specific cells compared to WT. Instead, enrichment of a terminal effector gene signature was lost in *Stat1*^T385M/+^ GP33-specific cells compared to WT (Fig. 2e). These results indicated that *Stat1* GOF expression results in T cell dysfunction rather than classical CD8 T cell exhaustion. As chronic antigen exposure is a key component driving T cell exhaustion^21^, viral load was measured to determine whether *Stat1*^T385M/+^ CD8 T cells can control LCMV-Arm infection. While one *Stat1*^T385M/+^ mouse had a high viral load, virus was otherwise controlled in *Stat1*^T385M/+^ mice like WT (Fig. 2f). Additionally, *Stat1*^T385M/+^ GP33-specific cells effectively upregulated Granzyme B expression with infection (Extended Data 1d-e), arguing that CD8 T cells from *Stat1*^T385M/+^ mice predominantly maintain their cytolytic activity and are not classically exhausted.

### The Integrated Stress Response (ISR) is active in T cells from *Stat1*^T385M/+^ mice

T cell hypo-responsiveness can exist along a heterogeneous continuum, and not all dysfunctional states represent irreversible exhaustion^22^. *Stat1*^T385M/+^ GP33-specific cells exhibited reduction of CD8 T cell despite acquisition of a Cell Cycle gene signature, Ki-67 expression and expected cycling state ratio (Fig. 3a and Extended Data 2a-c). We therefore sought to probe alternative mechanisms that underlie hypo-responsiveness of *Stat1*^T385M/+^ T cells. Notable enriched pathways include “*Cellular Responses to Stress”* as well as fundamental cellular processes like translation and metabolism (Fig. 3a), indicating that *Stat1*^T385M/+^ CD8 T cells experience increased cellular stress following LCMV-Arm infection.

The ISR is a process that enables cells to sense and adapt to diverse forms of stress, including the mentioned enriched pathways. Upon sensing various stress signals, cells activate the ISR pathway via phosphorylation of the translation initiation factor eIF2a (p-eIF2a), which reprograms transcription and translation to help restore homeostasis, promote survival, or trigger cell death (Fig. 3b)^23,24^. To investigate whether ISR is active in CD8 T cells from *Stat1*^T385M/+^ mice, we measured p-eIF2a and its downstream effects. Naïve and day 7 p.i. GP33-specific T cells from WT mice exhibited low p-eIF2a levels, whereas *Stat1*^T385M/+^ GP33-specific T cells showed significant induction (Fig. 3c). This increase was not specific to a particular subset, as elevated p-eIF2a was observed in bystander naïve *Stat1*^T385M/+^ CD8 T cells, antigen-specific CTLs, and memory precursors (MPEC) (Fig. 3d).

Phosphorylation of eIF2a leads to induced expression and translation of ATF4 which subsequently modulate the expression of stress response genes (Fig. 3b)^25^. Indeed, ATF4 transcript and protein were elevated in GP33-specific *Stat1*^T385M/+^ T cells compared to WT (Fig. 3e,f). Correspondingly, stress response and ATF4-target genes such as *Fos, Jun, Ppp1r15a* (encodes GADD34), and *Ddit3* (encodes CHOP) were upregulated in *Stat1*^T385M/+^ T cells (Fig. 3g).

A central consequence to an active ISR is stress granule formation and reduced cap-dependent translation (Fig. 3b)^23^. Stress granules are cytoplasmic ribosome-stalled mRNA complexes enriched for RNA-binding proteins such as G3BP1^26^. We found that WT CD8 T cells did not form stress granules during an effector response whereas *Stat1*^T385M/+^ T cells exhibited robust stress granule formation (Fig. 3h). However, GP33-specific *Stat1*^T385M/+^ cells also exhibited elevated translation measured by puromycin incorporation (Fig. 3i). This is consistent with a CD8 T cell proteotoxic stress response, in that cells exhibit increased protein translation, upregulation of chaperones, and formation of protein aggregates with G3BP1^27^. Indeed, GP33-specific *Stat1*^T385M/+^ cells also exhibited elevated expression of *G3bp1* and the chaperone *Hspa5* (encodes BiP) (Fig. 3j), indicative of *Stat1*^T385M/+^ cells also exhibiting features of proteotoxic stress^27^.

ISR can be a beneficial process to maintain cells during stress; however, if triggered and not reversed in time, cell death can ensue^24^. Indeed, we demonstrated that *Stat1*^T385M/+^ cells exhibited induction of an apoptotic gene program (Extended Data 2d-e) and increased pan caspase activity (Fig. 3k). Altogether, CD8 T cells from *Stat1*^T385M/+^ mice aberrantly present with an active ISR during the effector response with LCMV-Arm infection.

### CD8 T cell dysfunction in *Stat1*^T385M/+^ mice is cell extrinsic

We next examined whether CD8 T cells were also dysfunctional when *Stat1-*T385M expression was restricted to T cells via *CD4*-cre (referred to *CD4cre*^T385M/+^, pink throughout). Unexpectedly, *CD4cre*^T385M/+^ mice infected with LCMV-Arm did not result in viral lethality (Fig. 4a). Additionally, total CD8 T cells and GP33-specific CD8 T cells from *CD4cre*^T385M/+^ mice expanded comparably to WT mice after infection (Fig. 4b–e). Functionally, PD-1 expression was slightly increased in GP33-specific CD8 T cells from *CD4cre*^T385M/+^ mice; however, levels of LAG3, TIGIT, and Tim3 expression and cytokine production were comparable to WT CD8 T cells (Fig. 4f–h). Likewise, *CD4cre*^T385M/+^ CD8 T cells did not show elevated p-eIF2a, puromycin incorporation, or FAM-FLICA levels (Fig. 4i,j and Extended Data 3a), indicating ISR is inactive. Therefore, the major impact of *Stat1* GOF on CD8 T dysfunction and ISR activity is extrinsic.

### Intrinsic *Stat1*-T385M alters memory versus effector trajectories

While T cell-confined expression of *Stat1-*T385M (i.e., *CD4cre*^T385M/+^ mice) largely permitted normal CD8 T cell function (Fig. 4), we sought to determine if a STAT1-mediated transcriptomic program might be aberrant. We confirmed that *Stat1-*T385M expressing T cells continued to not generate normal proportions of terminal effector T cells compared to WT cells, most notably day 15 p.i. (Fig. 5a,b). Calculating the ratio of IL-7R^+^ -vs- KLRG1^+^ cells from WT and *CD4cre*^T385M/+^ mice revealed an altered differentiation trajectory, insofar as *CD4cre*^T385M/+^ cells exhibited a bias towards memory differentiation during infection (Fig. 5c). Likewise, scRNA+TCR-seq of GP33-specific *CD4cre*^T385M/+^ CD8 T cells revealed diminished frequency of KLRG1^+^CX3CR1^+^ terminal effector cells day 7 p.i. (Extended Fig 3b–d). Tracking TCR clones undergoing clonal expansion within WT GP33-restricted cells revealed a reduction in stem-like and intermediate populations accompanied by an increase in terminal effectors (Extended Data 3e), consistent with prior reports describing stepwise differentiation of PD-1^+^TCF-1^+^TOX^+^ stem-like CD8 T cells into terminal effector populations^28,29^. However, this transition did not occur in *CD4cre*^T385M/+^ mice (Extended Data 3e). Furthermore, in *CD4cre*^T385M/+^ cells, many TE genes (*Klrg1, Cx3cr1, Zeb2, Gzma,* and *S1pr5*) were reduced and memory-lineage genes (*Tcf7, CD27,* and *Tox*) were increased (Fig. 5d)^30^, demonstrating *Stat1* GOF alters the trajectory of CD8 T cells during acute viral infection.

CD8 T cell differentiation follows a coordinated progression in which antigenic stimulation and inflammatory cues shape transcriptional networks that determine cell fate. We next measured STAT1 activity to determine if *Stat1* GOF was promoting memory and/or hindering effector differentiation. Seven days post LCMV-Arm infection, *CD4cre*^T385M/+^ T cells showed elevated STAT1 phosphorylation (pSTAT1) ex vivo and more prominent STAT1 binding to chromatin compared to WT cells (Fig. 5e,f and Extended Data 4a). As expected, STAT1 bound stronger to interferon regulated genes in *CD4cre*^T385M/+^ T cells, such as *Tbx21, Stat1* and *Cxcr3* (Fig. 5h and Extended Data 4b). However, many terminal effector genes downregulated in *CD4cre*^T385M/+^ cells did not show ****differential STAT1 binding (e.g., *Klrg1, Cx3cr1, Zeb2, Gzma*; Fig. 5g and Extended Data 4c). This suggests that STAT1 has a limited and indirect impact on effector gene program. Instead, analysis of published perturbSEQ data identified T-bet (gene *Tbx21*) as a selective top upstream transcription factor that regulates gene expression differences in *Stat1* GOF CD8 T cells. Here, *Tbx21* deletion reduced WT DEGs, increased *CD4cre*^T385M/+^ DEGs, and exhibited increased STAT1 binding to its gene locus (Fig. 5h and Extended Data 4d,e). Likewise, this analysis showed Tcf-1 (*gene Tcf7*) and Lef-1 as additional top upstream factors that upon deletion increased WT DEGs and reduced *CD4cre*^T385M/+^ DEGs. STAT1 binding was also elevated in *CD4cre*^T385M/+^ cells at *Tcf7* and *Lef1* (Extended Data 4d-f). Together, these data suggest that *Stat1* GOF alters the balance of key transcription factors that determine effector versus memory differentiation.

STAT1 can induce and maintain T-bet expression, thereby regulating effector gene programs such as cytokine production, cytotoxic machinery, and effector differentiation^31^. However, *CD4cre*^T384M/+^ CD8 T cells with overactive STAT1 exhibited reduced effector differentiation and increased STAT1 binding to *Tbx21*, suggestive of *Stat1* GOF having a repressive effect on T-bet. Indeed, T-bet protein levels were reduced in terminal effectors from *CD4cre*^T385M/+^ mice (Fig. 5i). Numerous *Tbx21*-bound STAT1 peaks overlapped with ISRE motifs (Fig. 5h), favoring a model in which type I IFN-activated ISGF3 (Interferon-Stimulated Gene Factor 3) represses T-bet expression. Therefore, CD8 T cells from WT and *CD4cre*^T385M/+^ mice were stimulated with IFN-a ex vivo 7 days post LCMV-Arm infection to determine whether ISGF3/IFN-I represses T-bet expression. Both WT and *CD4cre*^T385M/+^ T cells exhibited reduced T-bet expression after IFN-a stimulation (Fig. 5j), demonstrating that STAT1 in the ISGF3 complex can restrain T-bet expression. Together, intrinsic *Stat1* GOF alters memory versus effector trajectories through IFN-I/ISGF3 mediated repression of T-bet.

### STAT1 directly regulates ISR genes in CD8 T cells

In CD4 T cells, ISR has emerged as a critical regulator of memory function, as it hinders the translation of cytokine encoding transcripts at baseline to prevent cytokine release prior to a secondary challenge^32,33^. We therefore sought to determine if published LCMV-Arm scRNA-seq datasets followed a similar trend in ISR-related gene expression (i.e. ISR Module). Indeed, the ISR module was low in naïve (day 0) and effector cells (day 8 p.i.) and increased in memory cells (days 15–30 p.i.) (Fig. 6a). ISR Module genes augmented in memory compared to naïve/effector cells included transcriptional effectors (*Atf4, Atf3*), factors that enable ISR recovery (*Ppp1r15a, Dnajb1*), and factors that regulate parallel stress responses (*Nfe2l2*) (Fig. 6b).

Considering *CD4cre*^T385M/+^ biases cells towards the memory trajectory, STAT1 could also regulate the ISR Module in CD8 T cells. Indeed, the ISR Module in effector GP33-specific cells exhibited a stepwise increase across genotypes, with *CD4cre*^T385M/+^ cells elevated relative to WT, and *Stat1*^T385M/+^ cells showing a further increase over *CD4cre*^T385M/+^ cells (Fig. 6c). However, some genes within the ISR Module exhibited selective preferential expression in *CD4cre*^T385M/+^ cells (e.g. *Ppp1r15a, Dnajb1*), with other genes only elevated in *Stat1*^T385M/+^ cells (e.g. *Atf5, Atf3, Ddit3*) (Fig. 6d). To determine whether STAT1 directly regulates ISR gene expression, we measured STAT1 binding to ISR genes. Most ISR Module genes were bound by STAT1 in both WT (blue) and *CD4cre*^T385M/+^ (dark red) GP33-specific cells 7 days post LCMV-Arm infection (Fig. 6d). Notably, STAT1 binding was higher in *CD4cre*^T385M/+^ cells compared to WT (Fig. 6e,f), suggesting that hyperactive STAT1 directly impacts ISR gene expression.

### Excessive type I IFN triggers and amplifies the ISR to promote apoptosis in CD8 T-lymphocytes

Our data argue that STAT1 can directly regulate ISR gene expression, which elicits divergent outcomes in *Stat1* GOF CD8 T cells depending on the genetic model studied. While CD8 T cells from *CD4cre*^T385M/+^ mice showed elevated ISR Module expression (Fig. 6) without a bona fide ISR response (Fig. 4), *Stat1*^T385M/+^ effector T cells had an active ISR with concomitant T cell dysfunction and increased apoptosis (Fig. 1, 3). This suggests that an extrinsic factor activates the ISR in *Stat1*^T385M/+^ mice. Leveraging a suite of complementary and unbiased analysis tools, IFN-Is were predicted to be the differential signal between ubiquitous and T-cell-specific *Stat1* GOF mice capable of inducing the ISR Module (Extended Data 5a-d); therefore, we measured IFN production across models. As expected, IFN-Is were ~10 pg/mL in WT mice 7 days p.i. (Fig. 7a); however, IFN-a and IFN-b were increased by 10-fold in *Stat1*^T385M/+^ mice (Fig. 7a), which also correlated positively with eIF2a phosphorylation (Fig. 7b). By comparison, IFN-g levels were similar between models (Fig. 7a), indicating protracted high IFN-I is likely responsible for an ISR in *Stat1*^T385M/+^ CD8 T cells. Indeed, WT and *CD4cre*^T385M/+^ mice administered recombinant mouse (rm) IFN-a showed a significant increase in p-eIF2a, ATF4, and translation after LCMV-Arm infection (Fig. 7c). Next, we determined whether ISR was reversible in *Stat1*^T385M/+^ mice by administering anti-IFNAR antibodies to block protracted IFN-I production. While we noted a trend towards p-eIF2a reduction, the response was variable and not significant (Extended Data 5e), suggesting that protracted IFN-I in *Stat1*^T385M/+^ mice triggers a cascade of events such that blocking IFN-I alone is no longer sufficient to reverse ISR activation. Altogether, excessive IFN-Is triggers the ISR in CD8 effector T cells.

Protein Kinase R (PKR) activates the ISR, and IFN-Is have been shown to both increase its expression and promote its phosphorylation via IFN-activated JAK kinases^34,35^. Therefore, PKR was examined to determine whether its activity was affected by elevated IFN-Is. As anticipated, p-PKR was elevated in GP33-specific T cells from *Stat1*^T385M/+^ mice compared to WT and *CD4cre*^T385M/+^ mice (Fig. 7d), and exogenous rm-IFN-a increased p-PKR in WT and *CD4cre*^T385M/+^ mice (Fig. 7e), favoring a model in which IFN-Is triggers the ISR in CD8 effector T cells through PKR.

Whether ISR activation leads to recovery or cell death depends on the intensity, duration, and context of the stress^24^. GADD34 (encoded by *Ppp1r15a*) and CHOP (encoded by *Ddit3*) are two downstream regulators of ISR outcome, where GADD34 facilitates recovery via eIF2a dephosphorylation, and CHOP is a pro-apoptotic transcription factor^24^. To determine whether IFNs promote or repress *Ppp1r15a* and *Ddit3* in WT settings, the Immune Dictionary dataset was interrogated^36^. At baseline, *Ppp1r15a* was expressed and *Ddit3* was absent in CD8 T cells (Fig. 7f). However, upon IFNa and IFNb administration, *Ppp1r15a* expression decreased and *Ddit3* expression increased (Fig. 7f), indicating that increased IFN-I levels biased CD8 T cells towards apoptosis. Likewise, 7 days after LCMV-Arm infection when IFN-I is protracted in *Stat1*^T385M/+^ mice, GP33-secific cells showed *Ddit3* induction compared to WT and *CD4cre*^T385M/+^ mice (Fig. 6d). Additionally, STAT1 bound directly to *Ddit3* (Fig. 6d), suggesting that STAT1 action through IFN-Is directly amplifies CHOP gene expression to promote cell death. Indeed, IFNa administration reduced CD8 T cell frequency and increased apoptosis in WT and *CD4cre*^T385M/+^ mice (Fig. 7g,h). Altogether, aberrant and protracted IFN-I production after acute viral infection leads to the ISR triggered in effector CD8 T cells and CHOP expression amplified that promotes cell death.

## DISCUSSION

STAT1 GOF syndrome is characterized by a concatenation of immune-mediated pathologies along with impaired host defense, with the STAT1-T385M variant conferring particularly severe clinical manifestations^13,37^. Despite enhanced IFN signaling, patients with STAT1 GOF and related interferonopathies paradoxically experience severe viral infection, underscoring the enigmatic complexities of IFNs^13,38–41^. Indeed, decades of research have established that IFN-Is exert dual, context-dependent effects on antiviral CD8 T cell immunity, capable of both promoting and constraining T cell responses. To dissect the consequences of enhanced STAT1 signaling, we utilized a tissue-specific mouse model in which STAT1-T385M expression was mediated by Cre recombination. Our findings reveal that STAT1 activity governed the balance between CD8 differentiation and cellular stress. Cell-intrinsic *Stat1* GOF limited effector differentiation and promoted the acquisition of memory-like gene program, including the expression of memory-associated stress response genes. In contrast, global *Stat1* GOF expression resulted in heightened and prolonged IFN-I responses, triggering activation of the integrated stress response (ISR) in effector CD8 T cells and cell death. Together, this study identifies a previously unappreciated diverse mechanisms of STAT1 in shaping CD8 T cell fate.

Consistent with our findings, a prior study similarly reported impaired T cell responses following gammaherpesvirus 68 infection, whereby *Stat1*-R274W mice exhibited reduced effector cell numbers, impaired cytokine production, and evidence of a memory bias^15^. Building on this, our study identifies an active ISR in dysfunctional *Stat1* GOF effector CD8 T cells. The ISR is a highly conserved normal physiological response that help cells respond to stress and has emerged as a critical regulator of memory CD4 T cells. ISR appears beneficial in memory T cells, where it uncouples memory differentiation from function^33^. For example, upon acquiring prominent cytokine mRNA levels, memory T cells utilize ISR to restrain cytokine translation^32^. Our data show a memory bias in *Stat1* GOF after infection, and STAT1 supports this by directly binding to and promoting ISR gene expression. These findings raise the possibility that a STAT1-ISR-memory axis may contribute to the spontaneous autoimmune manifestations seen in STAT1 GOF and IFN-associated diseases.

By contrast, our study indicates a sustained ISR is detrimental to effector CD8 T cell responses in *Stat1*^T385M/+^ mice. We found that the ISR module is low in WT effector CD8 T cells, and *bona fide* ISR activation arises specifically under conditions of excessive IFN-I–STAT1 signaling. However, with LCMV clone 13 that produces chronic IFN-I, exhausted CD8 T cells do not exhibit a prominent ISR signature^42^. This raises the possibility that stressed effector CD8 T cells from acute infection may preferentially undergo apoptosis instead of differentiating towards exhaustion, and/or exhausted T cells from chronic infection are able to resolve IFN-triggered stress. Additionally, these observations suggest that ISR activation alone is insufficient to drive exhaustion. Rather, stressed CD8 T cells may be more of a ‘cell state’ instead of a lineage, as seen by scRNA-seq of *Stat1* GOF T cells in which ISR impacts multiple clusters. This may be why exhausted CD8 T cells from tumors exhibit an active ISR^42–44^, which is likely due to the unique stressors found in a tumor microenvironment. Collectively, these observations suggest that ISR engagement is not a fixed checkpoint along a trajectory to exhaustion, but rather a context-dependent response shaped by the tissue and inflammatory environment in which CD8 T cells encounter stress.

Prior studies have established intrinsic anti-proliferative and pro-apoptotic functions for STAT1 in CD8 T cells, as STAT1 deficiency enhances T-cell expansion and survival following IFN-Istimulation^45^. Surprisingly, despite elevated STAT1 activity, proliferation and survival were preserved in CD8 T cells from *CD4cre*^T385M/+^ mice. Instead, *Stat1* GOF primarily constrained effector differentiation among clonally expanded T cells through direct repression of *Tbx21* (encodes T-bet) at ISGF3-bound loci. This finding was unexpected because inflammatory signals generally promote effector differentiation, and STAT1 has been shown to directly induce T-bet expression^46–49^. Our data suggest, however, that the consequences of STAT1 activation depend on the signaling context, with type I IFN–ISGF3 activity exerting effects distinct from IFN-γ-induced STAT1 homodimers. In this regard, our findings are consistent with observations from chronic viral infection, where prolonged IFN-I signaling is associated with reduced T-bet expression and impaired effector differentiation^50^. Notably, T-bet can also suppress the expression of IFN-I-responsive genes, indicating a reciprocal antagonism between T-bet and IFN-I– ISGF3 signaling^51^.

Beyond their canonical roles in antiviral defense and immune activation, IFNs also contribute to the immunopathology of cancer, infection, and autoimmune/autoinflammatory disease. Our data provide insight into this duality, whereby the same IFN-ISR axis that protects cells during the innate antiviral response has the opposite effect when engaged later. While IFNs induce and phosphorylate PKR to initiate the ISR cascade during the innate phase to restrict the translation of viral transcripts^23,34^, we show here that when engaged later, aberrant IFN-I–PKR–ISR activation in effector T cells culminates in T cell death. This shift from a protective innate function to a deleterious adaptive one may help explain why IFN-I therapy is not uniformly beneficial after viral infection. More broadly, it raises the possibility that the IFN-ISR axis contributes to immune dysfunction across interferon-mediated pathologies. Future work is needed to define the contexts in which IFN drives ISR activation toward protective versus pathogenic outcomes in these diseases.

## ONLINE METHODS

### Mice

*Stat1* conditional knock in mice were described previously^14^ and will be available at the Jackson Laboratory Repository (JAX stock No. 041102). CMV-Cre (no. 006054; B6.C-Tg(CMV-cre)1Cgn/J) and CD4-Cre (no. 022071; B6.Cg-Tg(Cd4-Cre)1Cwi/BfluJ) mice were purchased from JAX. All mice were kept on the C57BL/6 background and were housed and bred at the National Institutes of Health (NIH) in accordance with the guidelines of the Institutional Animal Care and Use Committee (protocol number A026–03-02). All mice used were 8 to 14 weeks of age. Both female and male mice were used without a sex bias apparent.

### Infection, anti-IFNAR blocking, and recombinant IFN-a treatment

Naïve mice were infected with 2 × 10^5^ LCMV Armstrong (Arm) and 2 × 10^6^ LCMV clone 13 by intravenous injection. For IFNAR blocking, mice were administered 300 mg of anti-IFNAR1 (clone MAR1–5A3, BioXCell #BP0241) intraperitoneally on days 5.5 and 7 post LCMV-Arm infection. For IFN-a treatment, mice were administered 1 mg of recombinant mouse IFNAA (R&D Systems, #10150-IF) intravenously or intraperitoneally on days 5.5 and 7 p.i..

### Flow Cytometry

All flow cytometry experiments are of lymphocytes isolated from uninfected mice or ex vivo after LCMV infection without additional stimulation, unless indicated. After cell-surface and fixable viability staining of single-cell suspensions with fluorophore-conjugated antibodies in PBS, intracellular staining was performed after fixing and permeabilizing cells with the Foxp3/transcription factor staining kit (eBioscience, #00–5523-00) according to the manufacturer’s instructions. For measuring levels of phosphorylated Stat, single-cell suspensions were stained with cell-surface fluorophore-conjugated antibodies and fixable viability dye in PBS, fixed for 20 min at 4°C with BD Fix/Perm solution (#554722), permeabilized with 100% cold methanol for 20 min at 4°C, and stained with anti-pStat fluorophore-conjugated antibodies in 1× Perm/Wash Buffer (BD Biosciences, #557885) for 30 min at room temperature. Flow cytometry antibodies and viability dyes used are described in detail in Supplementary Table 1. D^b^ GP33^+^ tetramer was provided by the NIH Tetramer Core Facility. Stained cells were acquired on a five-laser Cytek Aurora and analysis was performed with FlowJo v10.

### Puromycin assay

Isolated cells were incubated with Puromycin at 37°C in complete RPMI for 30 min. After surface and viability staining, cells were fixed and permeabilized using Biolegend’s Cyto-Fast Fix/Perm Buffer set (#426803) for 20 min and stained intracellularly with anti-Puromycin (clone 2A4) for 30 min prior to flow cytometric analysis.

### FAM-FLICA assay

To measure active caspase activity as a proxy for cell death, the Vybrant FLICA Caspase Apoptosis Assay kit (#V35117) was used according to the manufacturer’s instructions. Isolated cells were incubated with the FLICA substrate at 37°C in complete RPMI for 2 hrs prior to flow cytometric analysis.

### Confocal super-resolution microscopy

Confocal microscopy images of cells were obtained using a Zeiss LSM980 system equipped with the AiryScan2 detector (Zeiss). 16-bit micrographs were captured using the LSM modality of the ZEN3.6 software (Zeiss) to achieve sub-diffraction resolution and utilizing a Plan Apochromatic HC 63X/1.4 numerical aperture, oil immersion lens. Pinhole diameter was automatically set (typically 0.8–1.0 Airy units) while line averaging was consistently set to 2–4. Zoom was set to 4–5X throughout the imaging. Illumination of the samples was achieved using solid-state diode lasers with the following wavelengths: 405nm for Hoechst to mark the nuclei and 561nm to excite the AlexaFluor 568 secondary antibody (ThermoFisher Scientific, #A-11004) to mark G3BP1 (Cell Signaling Technology, #48372). Laser power and gain were adjusted to avoid pixel saturation and were left unchanged between samples to ensure reliable comparison between the two genotypes. Figure montages depict two-dimensional maximum projections obtained from the ZEN software after Z-stack acquisitions performed by setting optical sectioning along the Z axis at 0.5um thickness minimum. Micrographs were saved as .czi and then converted into uncompressed TIFFs. Linear adjustments of levels and cropping (when needed), measurements of granule volumes were performed on Imaris 11.0 (Bitplane) and montages were assembled using Adobe Photoshop 2026 and saved as TIFF with levels ungrouped.

### scRNA-seq and TCR-seq

Seven days after LCMV-Arm infection, single-cell suspensions from spleens of WT, *Stat1*^T385M/+^, and *CD4cre*^T385M/+^ mice were stained with allophycocyanin (APC)–conjugated GP33 tetramer (NIH Tetramer Facility) and magnetically enriched using MojoSort Mouse anti-APC beads (Biolegend, #480072) according to manufacturer’s protocol. Biological replicates stained with Total-Seq Hashtag antibodies (Supplemental Table 2) and mixed prior to loading into a Chromium controller, with one capture for each genotype. Library preparation was performed with Chromium GEM-X Universal 5’ Gene Expression v3 kit (10x Genomics), Chromium Single Cell Mouse TCR Amplification kit (10x Genomics), and Chromium GEM-X Single Cell 5 Feature Barcode kit v3 (10x Genomics) per manufacturer’s instructions. Purified libraries were sequenced on NovaSeq X Plus (Illumina). Alignment, filtering, barcode counting, unique molecular identifier counting, demultiplexing of hashtags, and TCR clonotype identity were performed using CellRanger (v9.0.1) with mkfast and multi. The GEX data were aligned to the mm10 reference genome, whereas hashtag oligos (HTO) data were processed using a custom feature reference file. Data were further analyzed using R (v4.5.2), Seurat (v5.4), and scRepertoire^52^.

For Seurat analysis, all samples were merged with Seurat’s merge function and filtered on the basis of nCount_RNA > 2000, nFeature_RNA > 1000, percentage of mitochondrial genes < 5. Gene expression was processed with the SCTransform, RunPCA, IntegrateLayers (method RPCAIntegration), FindNeighbors, FindClusters, and RunUMAP functions. Non-CD8 T cells were removed and reprocessed as mentioned, but variable genes that matched “^Tr.v”, “Îg.v”, “Lars2”, and “Gm43418” were removed from clustering analysis. Clusters were annotated by top cluster genes identified with FindAllMarkers and conventional markers. Differentially expressed genes (DEGs) were determined with FindMarkers (test.use = DESeq2) on pseudobulk populations generated with AggregateExpression. Pathway analysis was performed with Metascape^53^. Heatmaps were generated with Pheatmap on the terminal effector cluster that was pseudobulked with AverageExpression. GSEA analysis was performed with fgsea (v1.36.2), with CTL and Terminal Exhaustion genesets identified from^19^. To infer which cytokines drive changes in gene expression, we performed Immune Response Enrichment Analysis (IREA; https://www.immune-dictionary.org/app/home), the companion software for the Immune Dictionary which implements statistical tests to assess the enrichment of cytokine signatures in transcriptomes^36^. DEGs from *Stat1*^T385M/+^ and *CD4cre*^T385M/+^ cells were input into IREA to identify enriched cytokine response signatures and cell polarization states. Transcription Factor Enrichment analysis was performed by the ChIP-ATLAS online tool (chip-atlas.org)^54^, where DEGs from *Stat1*^T385M/+^ and *CD4cre*^T385M/+^ cells were input for dataset A and dataset B, respectively.

For scRepertoire analysis, filtered_contig_annotations files generated with CellRanger were used at the input file for clonotype analysis. Clonotype files were combined with the Seurat analysis, and clonotype expansion was determined with combineExpression (cloneCall = “strict”, cloneSize = c(Single=1, Small=5, Medium=20, Large=100, Hyperexpanded=500)).

### CUT&TAG

Single-cell suspensions were generated from spleens of WT and *CD4cre*^T385M/+^ mice 7 days post LCMV-Arm infection. Cells were stained with APC–conjugated GP33-tetramer (NIH Tetramer Facility) and isolated using MojoSort Mouse anti-APC beads (BioLegend, #480072) according to the manufacturer’s protocol. Isolated cells were next used for CUT&Tag (cleavage under targets and tagmentation). CUT&Tag was performed with Active Motif ’s CUT&Tag-IT Assay kit (anti-rabbit, #53171) as per the manufacturer’s protocol. Five hundred thousand cells were used for each immunoprecipitation with antiStat1 (1:50; Cell Signaling Technology, #14994), and immunoglobulin G (1:50; Cell Signaling Technology, #663262). CUT&Tag was performed with biological duplicates. PCR amplification was performed with 13 cycles. Purified libraries were sequenced on NextSeq2000.

Paired-end reads were quality controlled with FastQC and aligned to mm10 via Bowtie2, and peaks were called with Sparse Enrichment Analysis for CUT&RUN (SEACR)^55^ using IgG as a control to generate an empirical threshold for peak calling. Overlapping peaks between biological replicates were determined with the intersect command from the BEDtools package^56^ and retained for downstream analysis. Annotation of peaks was determined with annotatePeaks.pl from HOMER^57^. Differential STAT1 binding was identified with DESeq2 following the protocol (Zheng Y el al., 2020, protocols.io)^58,59^. Peaks were considered differentially bound between samples with Log_2_ fold change > 0.5, p-value < 0.05, and baseMean > 50. Visualizations include genomic tracks of STAT1 bigwig files of one biological replicate with using Integrative Genomics Viewer (https://igv.org/). The number of overlapping peaks between WT and *CD4cre*^T385M/+^ GP33-specific cells was determined with intersect from the BEDtools package.

### Viral quantification

LCMV-Arm viral titers were determined as previously described^60^. RNA was isolated from 25ul of serum using Zymo Direct-zol RNA Micropret kit with DNase I treatment (Zymo Research, #R2060). After isolation, the RNA concentration was measured using Nanodrop for each sample, and cDNA was generated with SuperScript III Reverse Transcriptase (ThermoFisher Scientific, #18080044). cDNA was added into a mastermix containing PowerUP SYBR Green mix (appliedBiosciences) and primers specific to LCMV GP (forward: CATTCACCTGGACTTTGTCAGACTC; reverse: GCAACTGCTGTGTTCCCGAAAC). Copy number was determined by comparing cycle threshold (Cq) values to a standard curve of known dilutions of a LCMV plasmid (addgene #15796).

### Measurement of serum interferon levels

Sera were collected by isolating whole blood, rested at room temperature for 30 min for coagulation, and centrifuged at 3000g for 20 min. Serum was stored at −80°C. To quantify interferon levels, the BioLegend LEGENDplex mouse Mouse Type 1/2 Interferon panel (Biolegend, #740635) was used according to the manufacturer’s protocol.

### Statistical analysis

Statistical analysis was performed with Prism (GraphPad, v11.0.2) and R (v4.5.2). Statistical significance between two independent experimental conditions was determined by unpaired two-tailed Student’s t test. Survival curve analysis was determined by log-rank (Mantel-Cox) test. Mean comparison for transcript levels and scRNA-seq modules scores were made with the stat_compare_means function (method = wilcox.test) in ggpubr (v0.6.0). Data are shown as means ± SE. Nomenclature for significance is as follows: *P < 0.05, **P < 0.01, ***P < 0.001. and ****P < 0.0001. Dots in graphs represent individual experimental animals.

## Supplementary Material

Supplementary Files

This is a list of supplementary files associated with this preprint. Click to download.

20260811S1gofT8FigsSuppl.docx

## Figures and Tables

**Figure 1:**
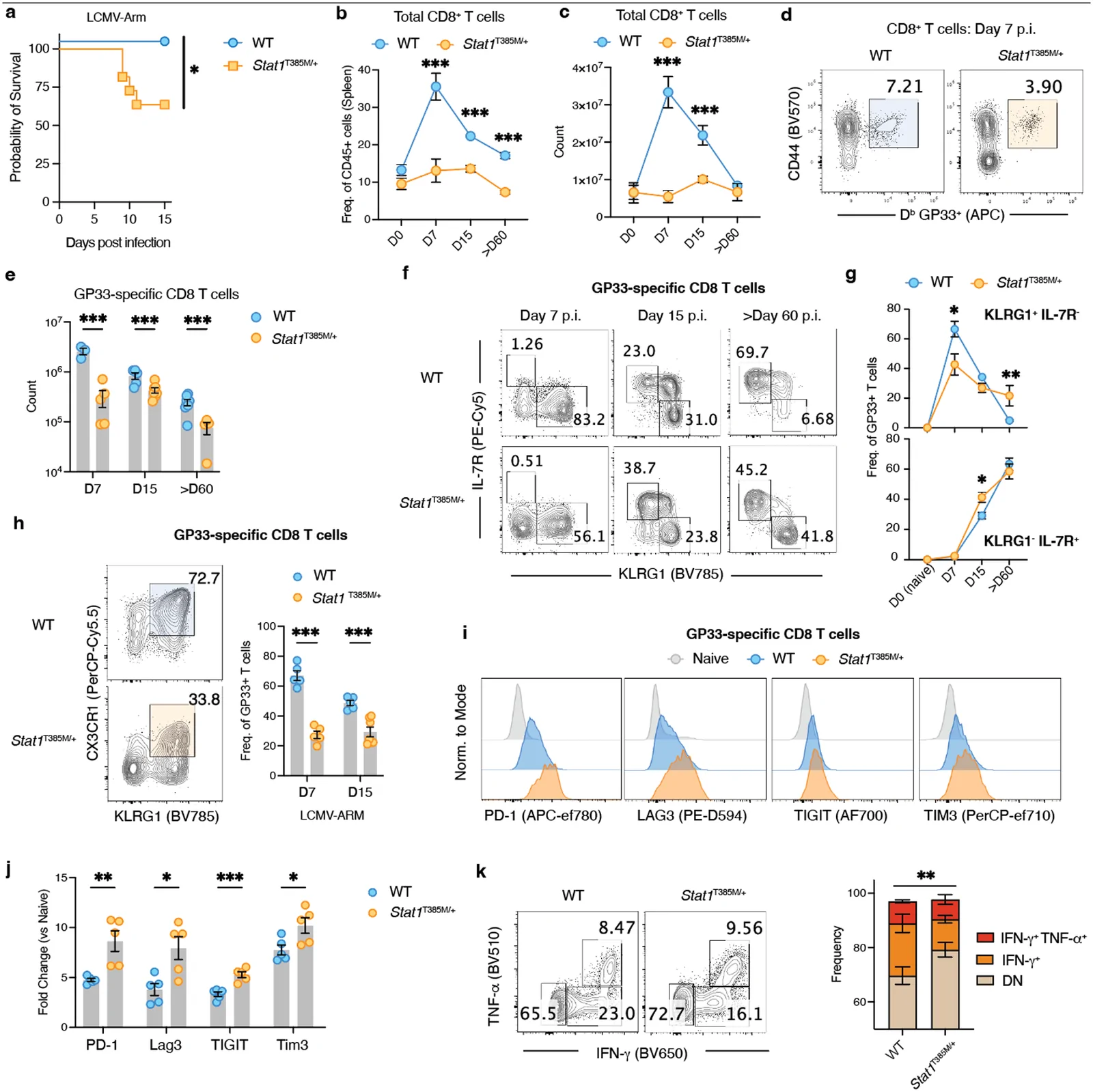
CD8 T cells from *Stat1*^T385M/+^ mice are dysfunctional after acute viral infection All *Stat1*^*T385M*/+^ mice have ubiquitous (via CMVcre) and heterozygous Stat1-T385M expression. **a**, Survival curve of WT and *Stat1*^T385M/+^ mice infected with LCMV-Armstrong (Arm), with *n* = 14 WT and *n* = 17 *Stat1*^T385M/+^ mice pooled from three independent experiments. **b-c**, Frequency (b) and cell count (c) of CD8^+^ T cells from the spleen after LCMV-Arm infection, with *n* = 4–7 WT and *n* = 4–8 *Stat1*^T385M/+^ mice pooled from five independent experiments. **d**, Representative flow cytometry plots of D^b^ GP33^+^-tetramer-positive CD8 T cells 7 days post LCMV-Arm infection. **e**, Cell count of GP33-specific CD8 T cells at 7, 15 and >60 days post LCMV-Arm infection, with *n* = 3–7 WT and *n* = 4–7 *Stat1*^T385M/+^ mice pooled from five independent experiments. **f-g**, Representative flow plots (f) and pooled data (e) showing the frequency of IL-7R^−^KLRG1^+^ and IL-7R^+^KLRG1^−^ GP33-specific CD8 T cells at 7, 15 and >60 days post LCMV-Arm infection, with *n* = 4–7 WT and *n* = 4–8 *Stat1*^T385M/+^ mice from five independent experiments. **h**, Frequency of CX3CR1^+^KLRG1^+^ GP33-specific CD8 T cells at days 7 and 15 post LCMV-Arm infection, with *n* = 5–7 mice per group pooled from three independent experiments. **i**, Representative flow cytometry histograms of PD-1, LAG3, TIGIT, and TIM3 expression in naïve CD8 T cells from uninfected WT mice and GP33-specific CD8 T cells from WT and *Stat1*^T385M/+^ mice day 7 post LCMV-Arm infection. **j**, Plots depict expression of PD-1, LAG3, TIGIT, and TIM3 in WT and *Stat1*^T385M/+^ GP33-specific CD8 T cells relative to naïve CD8 T cells from uninfected WT mice. *n* = 3–5 mice pooled from two independent experiments. **k**, Frequency of IFN-g and TNF-a producing CD44^+^ CD8 T cells after GP33 peptide stimulation in vitro, with *n* = 4–5 mice pooled from two independent experiments. Significance was determined with log-rank (Mantel-Cox) test for survival and unpaired Student’s t test for pairwise comparisons. *P < 0.05, **P < 0.01 and ***P < 0.001.

**Figure 2:**
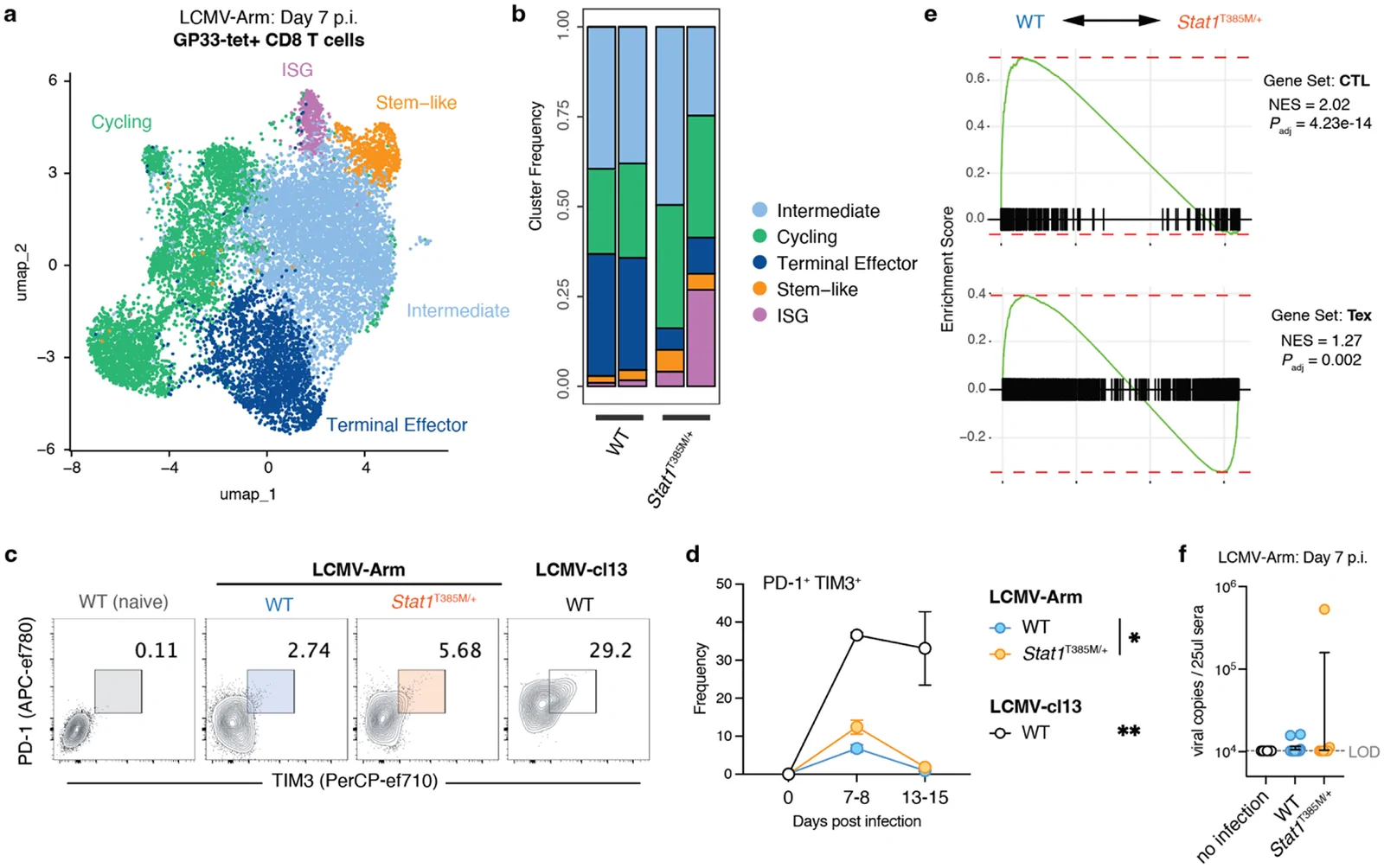
CD8 T cells from *Stat1*^T385M/+^ mice are not terminally exhausted Significance for PD-1^+^TIM3^+^ plots was determined by 2-way ANOVA, comparing WT and *Stat1*^T385M/+^ mice after LCMV-Arm infection, and WT-LCMV-Arm with WT-LCMV-cl13 infection. *P < 0.05 and **P < 0.01. **a**, UMAP projection and clustering identification of scRNA-seq data enriched from D^b^ GP33^+^-tetramer-positive CD8 T cells day 7 post LCMV-Arm infection. Cluster names were determined by top marker genes (Extended Data 1a). UMAPs depict merged genotypes. **b**, Proportions of scRNA-seq clusters from WT and *Stat1*^T385M/+^ mice, with *n* = 2 mice per group. **c**, Representative flow cytometry plots depicting the frequency of PD-1^+^TIM3^+^ cells of naïve WT CD8 T cells from uninfected mice, GP33-specific CD8 T cells from WT and *Stat1*^T385M/+^ mice day 7 post LCMV-Arm infection, and GP33-specific WT CD8 T cells day 7 post LCMV clone 13 (cl13) infection. **d**, Plots depict frequency of PD-1^+^TIM3^+^ cells as described in (c) at days 7–8 and days 13–15 post infection. *n* = 4 WT (naïve), *n* = 4–8 mice per group with LCMV-Arm infection, and *n* = 2–4 mice with LCMVcl13 infection, pooled from at least two independent experiments. **e**, Gene set enrichment analysis (GSEA) illustrating the enrichment of cytotoxic lymphocytes (CTL) genes and terminally exhausted cells (Tex) genes between WT and *Stat1*^T385M/+^ GP33-specific cells. Normalized enrichment score (NES) and adjusted *P* values (*P*_adj_) are indicated. **f**, Viral copy number in the sera from uninfected WT mice and LCMV-Arm infected WT and *Stat1*^T385M/+^ mice day 7 post infection, measured by RT-qPCR. *n* = 6–14 mice per group pooled from two independent experiments. Limit of detection (LOD) by a standard curve is indicated.

**Figure 3:**
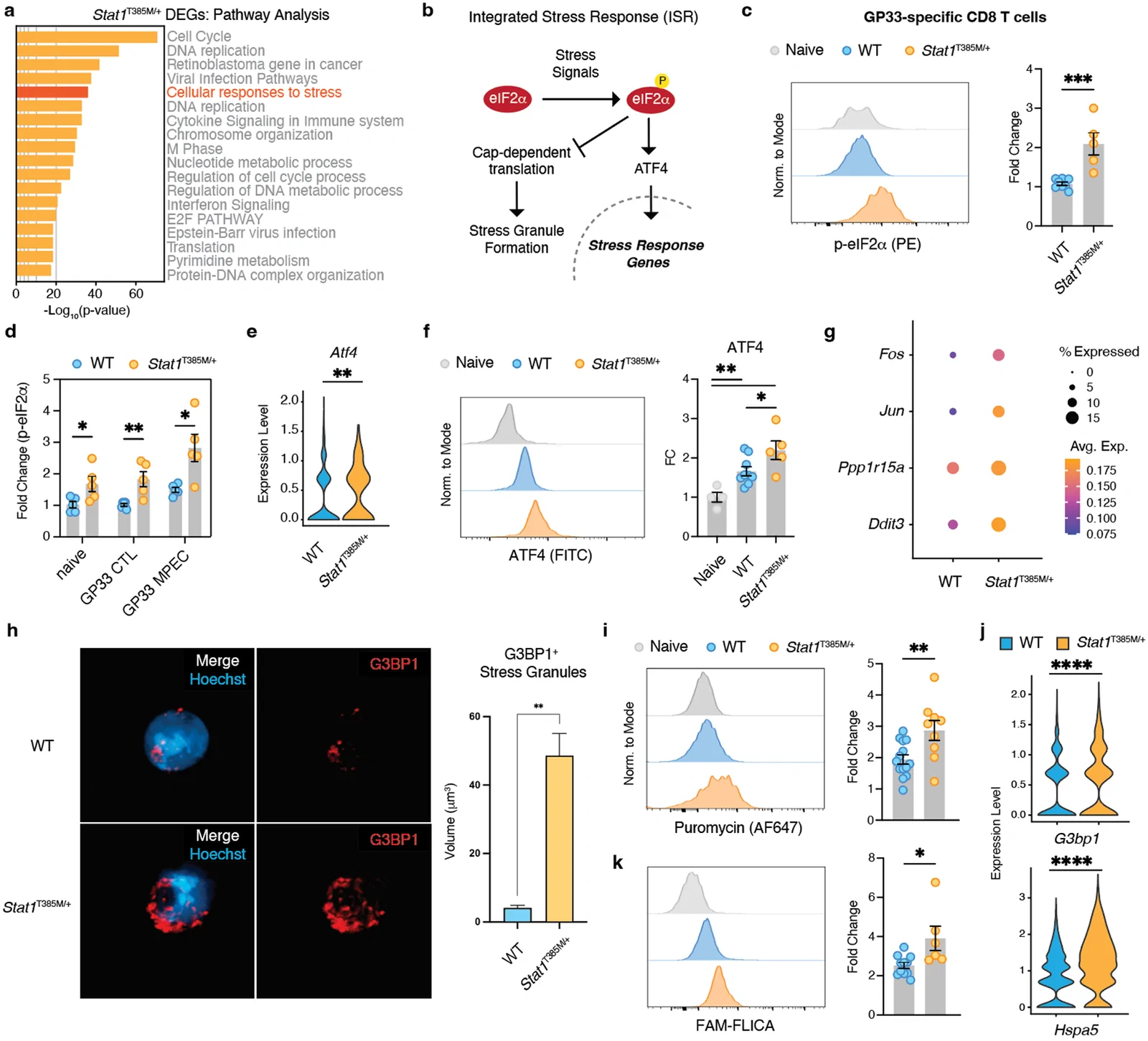
The integrated stress response (ISR) is active in T cells from *Stat1*^T385M/+^ mice **a**, Pathway enrichment analysis of *Stat1*^T385M/+^ DEGs performed with Metascape. **b**, Diagram of the Integrated Stress Response (ISR) pathway. **c**, p-eIF2a levels measured ex vivo by flow cytometry in naïve CD8 T cells from uninfected WT mice, and GP33-specific CD8 T cells from WT and *Stat1*^T385M/+^ mice 7 days post LCMV-Arm infection. Bar plots depict relative levels of GP33-specific cells over WT naïve cells, with *n* = 8 WT and *n* = 5 *Stat1*^T385M/+^ mice pooled from two independent experiments. **d**, Relative p-eIF2a levels in CD8 T cell subsets from WT and *Stat1*^T385M/+^ mice 7 days post LCMV-Arm infection. Subset depicted are naïve CD8 T cells, cytotoxic lymphocytes (CTL; CX3CR1^+^KLRG1^+^ of D^b^GP33^+^ cells) and memory precursor effector cells (MPEC; IL-7R^+^KLRG1^−^ of D^b^GP33^+^ cells), with *n* = 5 mice per group pooled from two independent experiments. **e**, *Atf4* transcript expression in the terminal effector cluster from Fig. 2a. **f**, ATF4 protein expression of GP33-specific CD8 T cells day 7–8 post LCMV-Arm infection, measured ex vivo by flow cytometry. *n* = 4–9 mice per group pooled from three independent experiments. **g**, Transcript levels of the ISR stress response genes *Fos*, *Jun*, *Ppp1r15a*, and *Ddit3* in the terminal effector cluster from Fig. 2a. **h**, Confocal images of GP33-specific CD8 T cells from WT and *Stat1*^T385M/+^ mice 7 days post LCMV-Arm infection, labelling G3BP1 for stress granules and Hoechst for nuclei. Bar plot quantifies volume of G3BP1^+^ stress granules from *n* = 8 WT cells and *n* = 5 *Stat1*^T385M/+^ cells. **i**, Representative histograms (left) and quantification (right) anti-puromycin geometric Mean Fluorescence Intensity (gMFI) in primary cells cultured ex vivo for 30 minutes with puromycin. Bar plots depict relative levels of GP33-specific cells over WT naïve cells from uninfected mice, with *n* = 8 mice per group pooled from four independent experiments. **j**, *G3bp1* and *Hspa5* transcript expression in the terminal effector cluster from Fig. 2a. **k**, Representative plots (left) and quantification (right) of pan-caspase activation at day 7–8 post infection, with *n* = 6–7 mice per group pooled from three independent experiments. Significance was determined with unpaired Student’s t test for pairwise comparisons of imaging and flow cytometry experiments, and Wilcoxon test for comparisons of scRNA-seq data. *P < 0.05, **P < 0.01, ***P < 0.001 and ****P < 0.0001.

**Figure 4:**
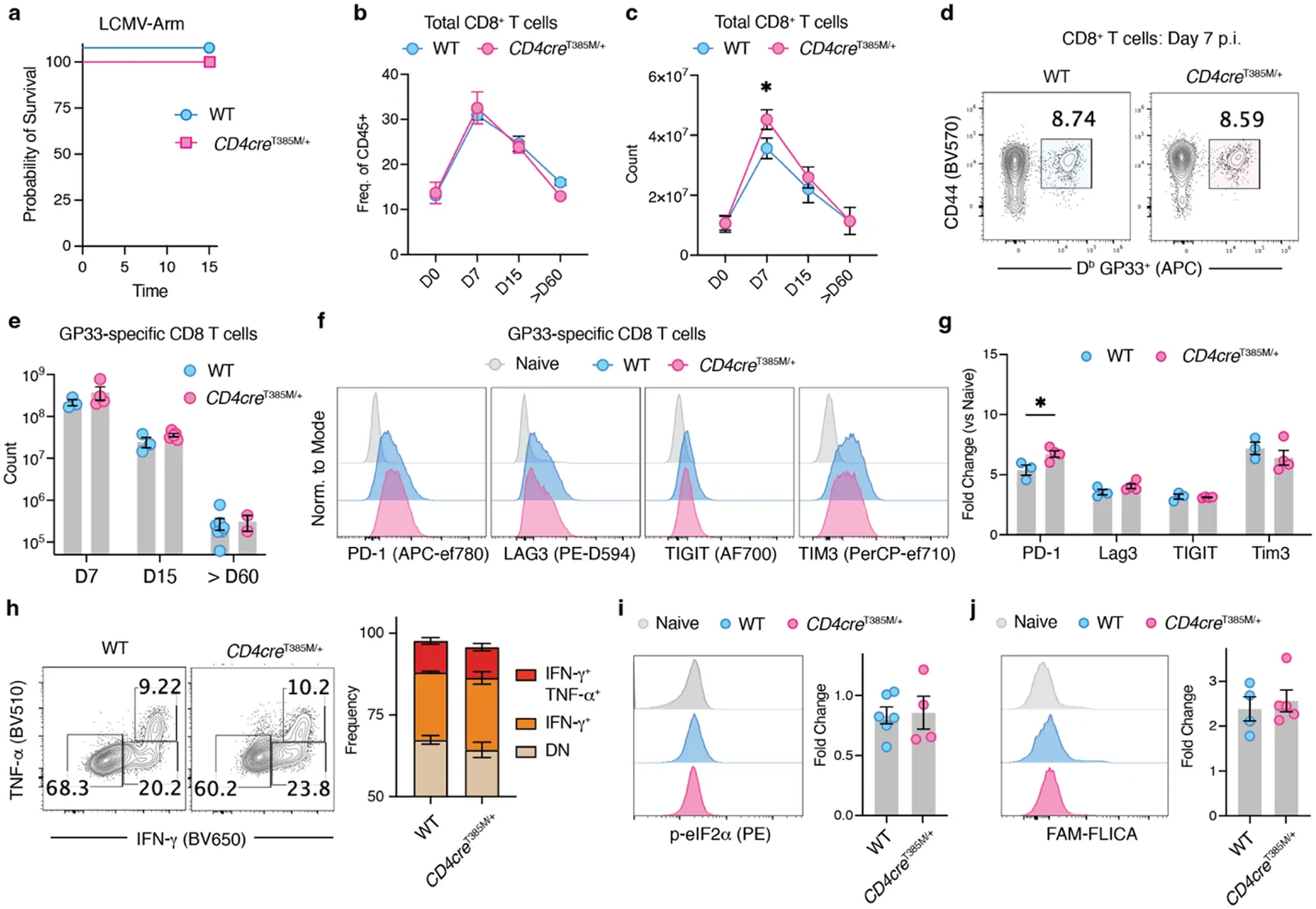
CD8 T cell dysfunction in *Stat1*^T385M/+^ mice is cell extrinsic All *CD4cre*^*T385M*/+^ mice have T-cell-restricted (via CD4cre) and heterozygous Stat1-T385M expression. **a**, Survival curve of WT and *CD4cre*^T385M/+^ mice infected with LCMV-Armstrong (Arm), with *n* = 10 WT and *n* = 9 *CD4cre*^T385M/+^ mice pooled from three independent experiments. **b-c**, Frequency (b) and cell count (c) of CD8^+^ T cells from the spleen after LCMV-Arm infection, with *n* = 3–8 WT and *n* = 2–8 *CD4cre*^T385M/+^ mice pooled from six independent experiments. **d**, Representative flow cytometry plots of D^b^ GP33^+^-tetramer-positive CD8 T cells 7 days post LCMV-Arm infection. **e**, Cell count of GP33-specific CD8 T cells at 7, 15 and >60 days post LCMV-Arm infection, with *n* = 2–7 mice per group pooled from at least two independent experiments. **f**, Representative flow cytometry histograms of PD-1, LAG3, TIGIT, and TIM3 expression in naïve CD8 T cells from uninfected WT mice and GP33-specific CD8 T cells from WT and *CD4cre*^T385M/+^ mice day 7 post LCMV-Arm infection. **g**, Plots depict expression of PD-1, LAG3, TIGIT, and TIM3 in WT and *CD4cre*^T385M/+^ GP33-specific CD8 T cells relative to naïve CD8 T cells from uninfected WT mice. *n* = 3–4 mice pooled from two independent experiments. **h**, Frequency of IFN-g and TNF-a producing CD44^+^ CD8 T cells after GP33 peptide stimulation in vitro, with *n* = 5–7 mice pooled from two independent experiments. **i**, Representative plots (left) and quantification (right) of p-eIF2a levels at day 7–8 post infection, with *n* = 4–6 mice per group pooled from three independent experiments. **j**, Representative plots (left) and quantification (right) of pan-caspase activation at day 7–8 post infection, with *n* = 4–5 mice per group pooled from three independent experiments. Significance was determined with log-rank (Mantel-Cox) test for survival and unpaired Student’s t test for pairwise comparisons. *P < 0.05.

**Figure 5:**
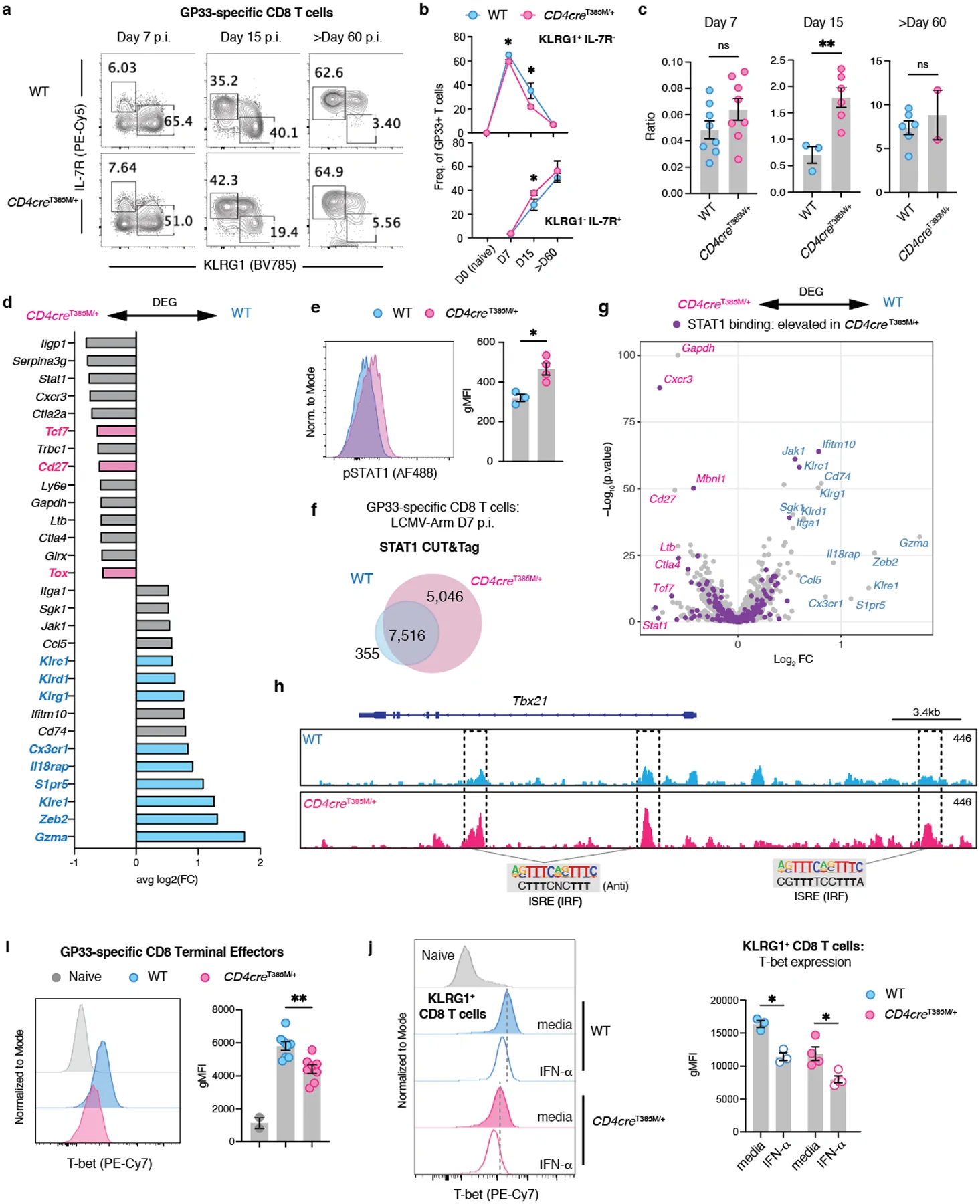
Intrinsic *Stat1*-T385M alters memory versus effector trajectories **a-b**, Representative flow plots (a) and pooled data (b) showing the frequency of IL-7R^−^KLRG1^+^ and IL-7R^+^KLRG1^−^ GP33-specific CD8 T cells at 7, 15 and >60 days post LCMV-Arm infection, with *n* = 4–8 WT and *n* = 2–8 *CD4cre*^T385M/+^ mice from six independent experiments. **c**, Ratio of IL-7R^+^ to KLRG1^+^ populations in (b). **d**, Top 30 differentially expressed genes (DEGs) between WT and *CD4cre*^T385M/+^ GP33-specific CD8 T cells 7 days post LCMV-Arm infection. DEG analysis was performed on pseudobulk populations from scRNA-seq. Pink text identifies memory-lineage genes and blue text highlights effector-lineage genes. **e**, Representative flow cytometry plots and quantification of pSTAT1 levels ex vivo on 7 days post LCMV-Arm infection, with *n* = 3 WT and 4 *CD4cre*^T385M/+^ mice pooled from two independent experiments. **f**, On day 7 post LCMV-Arm infection, CUT&Tag for STAT1 binding to chromatin was performed on GP33-specific CD8 T cells from WT and *CD4cre*^T385M/+^ mice. Venn diagram indicates the number of unique and overlapping STAT1-binding peaks between mice. Peaks are those common between two biological replicates. **g**, Volcano plot of DEGs comparing GP33-specific T cells from WT and *CD4cre*^T385M/+^ mice. Purple dots indicate DEGs where STAT1 binding peaks are elevated in *CD4cre*^T385M/+^ cells compared to WT, and grey dots represent all remaining DEGs. **h**, Tracks of STAT1 binding at the *Tbx21* and *Tcf7* locus. Highlighted regions indicate peaks with ISRE motifs, which also exhibit changes in STAT1 binding in *CD4cre*^T385M/+^ cells. **i**, Representative flow plots and pooled data showing T-bet levels in naïve CD8 T cells from uninfected WT mice, and KLRG1^+^CX3CR1^+^ GP33-specific CD8 T cells from WT and *CD4cre*^T385M/+^ mice 7 days post LCMV-Arm infection. *n* = 7 mice per group of infected conditions pooled from two independent experiments. **j**, Day 7 post LCMV-Arm infection, splenocytes were stimulated with or without IFN-a in culture for 5 hours, and naïve and KLRG1^+^ CD8 T cells were evaluated for T-bet expression. *n* = 3–4 mice per group representative of two independent experiments. Significance was determined by unpaired student’s t test for pairwise comparisons of flow cytometry experiments. *P < 0.05 and **P < 0.01.

**Figure 6:**
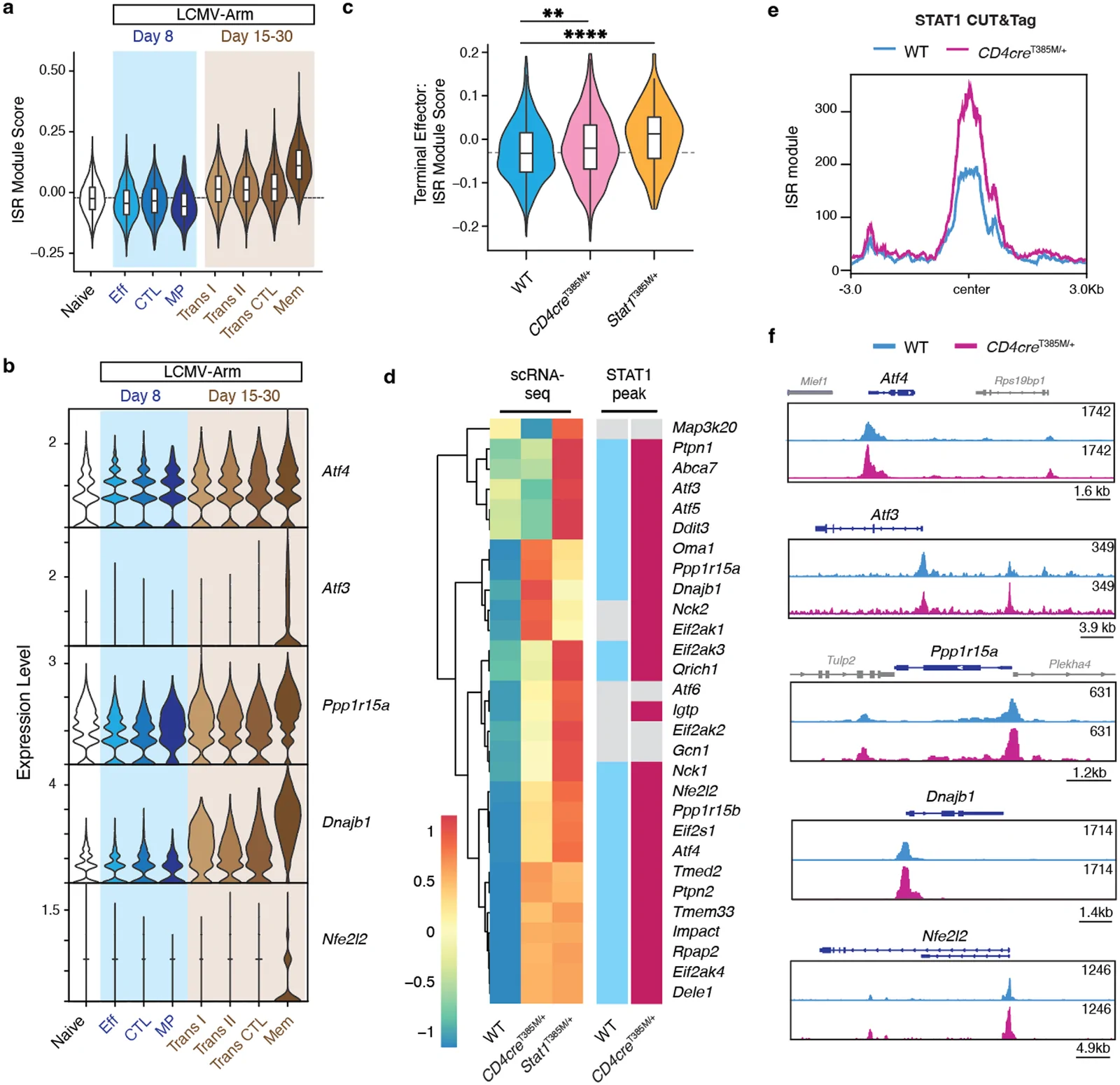
STAT1 directly regulates ISR genes in CD8 T cells **a**, Module score of ISR genes were measured in P14 CD8 T cells from LCMV-Arm infected mice, from the processed Seurat R object of the public dataset GSE199565, maintaining cluster identifiers from the original publication. **b**, Violin plots of example ISR genes from (a). **c**, Module score of ISR genes in terminal effector cells identified in Fig. 2a from WT, *CD4cre*^T385M/+^, and *Stat1*^T385M/+^ mice. **d**, ISR genes were evaluated for transcript levels and STAT1 binding, using terminal effector cells from Fig. 2a and STAT1 CUT&Tag from Fig. 5f, respectively. The gene expression heatmap is scaled on pseudobulk populations. The STAT1 peak grid identifies STAT1 binding in WT (blue) and *CD4cre*^T385M/+^ (maroon) cells, with grey boxes representing the absence of STAT1 binding. **e**, Average STAT1 signal from WT and *CD4cre*^T385M/+^ cells at peaks near ISR genes, within 1kb upstream of the TSS and 1kb downstream of TTS. Plots show signal +/− 3kb around the STAT1 peak. **f**, Tracks of STAT1 binding at example ISR genes. Significance was determined by paired Student’s t test for pairwise comparisons of flow cytometry experiment, and Wilcoxon test for comparisons of scRNA-seq data. *P < 0.05, **P < 0.01, and ****P < 0.0001.

**Figure 7:**
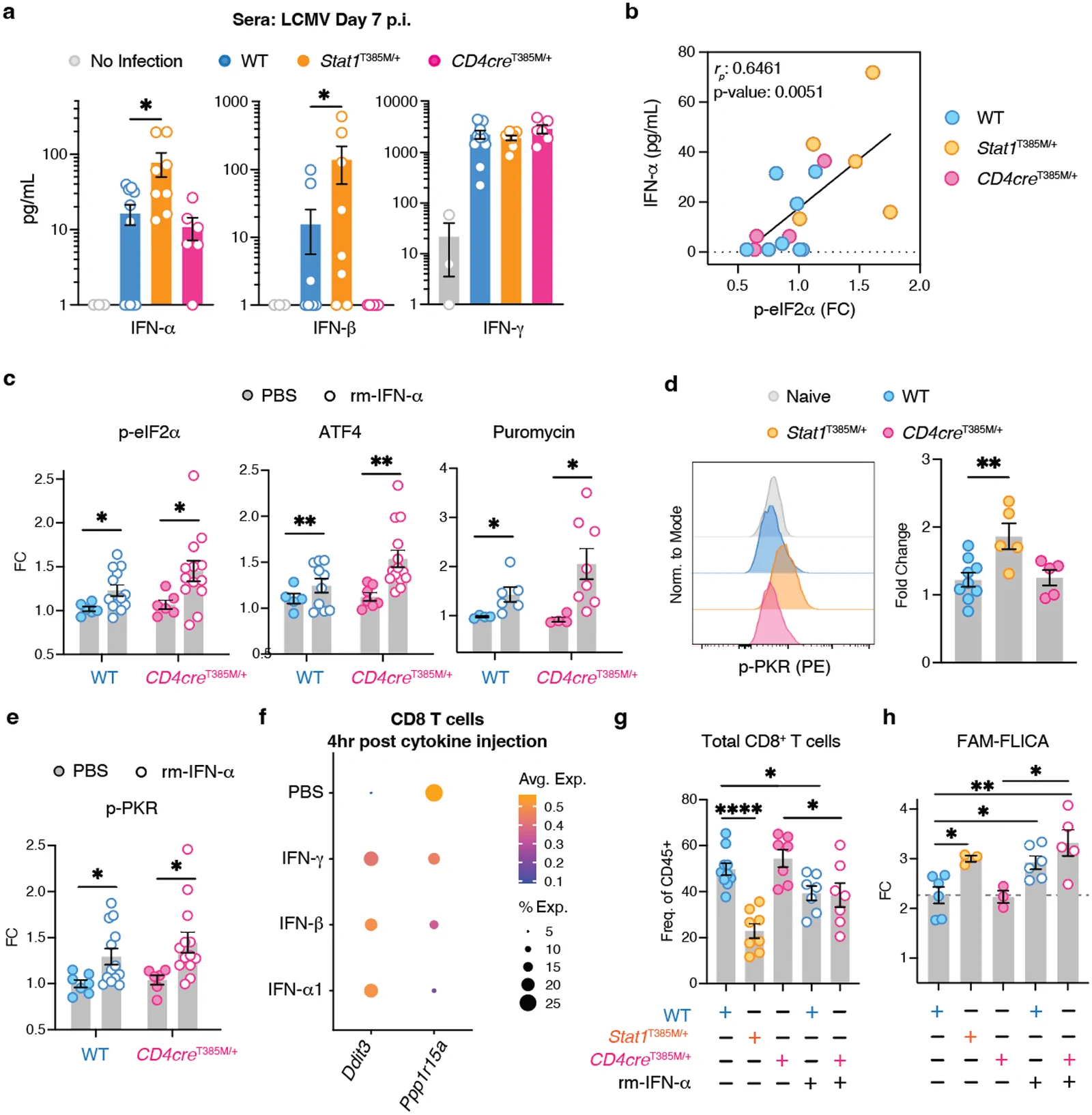
Excessive type I IFN triggers and amplifies the ISR to promote apoptosis CD8 T-lymphocytes **a**, Serum cytokine levels 7 days post infection from WT, *Stat1*^T385M/+^, and *CD4cre*^T385M/+^ mice. *n* = 6–11 mice per group of infected mice and *n* = 3 from WT uninfected mice, pooled from three independent experiments. **b**, Correlation of IFN-a and p-eIF2a levels measured from the same mouse. **c**, Normalized p-eIF2a, ATF4, and Puromycin levels day 7.5 post infection in GP33-specific CD8 T cells from WT and *CD4cre*^T385M/+^ mice after PBS or recombinant mouse (rm) IFN-a administration on days 5.5 and 7 post infection. *n* = 4–13 WT and *n* = 4–14 *CD4cre*^T385M/+^ mice pooled from 3–5 independent experiments. **d**, p-PKR levels measured ex vivo by flow cytometry in naïve CD8 T cells from uninfected WT mice, and GP33-specific CD8 T cells from WT, *Stat1*^T385M/+^, and *CD4cre*^T385M/+^ mice 7 days post LCMV-Arm infection. Bar plots depict relative levels of GP33-specific cells over WT naïve cells, with *n* = 5–9 mice per group pooled from three independent experiments. **e**, Normalized p-PKR levels day 7.5 post infection in GP33-specific CD8 T cells from WT and *CD4cre*^T385M/+^ mice after PBS or rm-IFN-a administration on days 5.5 and 7 post infection. *n* = 7–13 WT and *n* = 6–113 *CD4cre*^T385M/+^ mice pooled from five independent experiments. **f**, Expression of *Ddit3* (encodes CHOP) and *Ppp1r15a* (encodes GADD34) in WT CD8 T cells 4 hours post PBS and cytokine injection, from the processed Seurat R object of the public dataset GSE202186. **g**, Frequency of CD8^+^ T cells from the spleen after LCMV-Arm infection, with *n* = 7–10 mice/group pooled from five independent experiments. **h**, Normalized pan-caspase activation on day 7.5 post infection in GP33-specific CD8 T cells after indicated PBS or rm-IFN-a administration days 5.5 and 7 post infection. *n* = 3–6 mice per group pooled from three independent experiments. Significance was determined by Mann-Whitney for serum cytokine measurements, Pearson for correlation analysis, and unpaired Student’s t test for pairwise comparisons of flow cytometry experiments. *P < 0.05 and **P < 0.01.

## Data Availability

The scRNA/TCR-seq and STAT1 CUT&Tag data generated in this study are available under the accession number XXX. Publicly available datasets analyzed during this manuscript were downloaded from: dryad for perturbSEQ (https://doi.org/10.5061/dryad.s7h44j1gr)^61^, the Immune Dictionary portal (https://www.immune-dictionary.org/app/home)^36^, and GSE199565^19^.

## Code availability

This study did not generate any new code. All bioinformatic analyses were performed using publicly available packages and code with the parameters described in the Methods.
